# Fine mapping genetic variants affecting birth weight in sheep: a GWAS of 3007 individuals using low-coverage whole genome sequencing

**DOI:** 10.1186/s40104-025-01251-4

**Published:** 2025-08-12

**Authors:** Ran Li, Yuheng Bai, Maqiang Zhao, Xinyue Zhang, Haiyan Wang, Bo Feng, Shuo Zhang, Huanhuan Zhang, Gang Ren, Xihong Wang, Yu Jiang

**Affiliations:** 1 Key Laboratory of Animal Genetics, Breeding and Reproduction of Shaanxi Province, College of Animal Science and Technology, Northwest A&F University, Yangling, 712100 Shaanxi China; 2 Yulin Industrial Development Institute for Sheep & Goat, Yulin, 719053 Shaanxi China

**Keywords:** Birth weight, GWAS, LcWGS, QTL, Sheep

## Abstract

**Background:**

Birth weight is a critical economic trait in livestock production. However, its genetic architecture remains poorly understood due to historical limitations in sample size and reliance on low-density SNP arrays. In this study, we utilized low-coverage whole-genome sequencing (lcWGS) to genotype 3,007 Hu sheep, bypassing the cost and resolution constraints of conventional genotyping arrays while achieving scalable genome-wide variant detection.

**Results:**

LcWGS with high imputation accuracy (97.8% allelic concordance) enabled genome-wide association studies (GWAS) identifying two novel quantitative trait loci (QTLs) on chromosomes 6 and 9. The chromosome 9 QTL encompassed a regulatory region functionally linked to *PLAG1* expression through expression quantitative trait locus (eQTL) mapping. Compared with wild-type homozygotes, heterozygous carriers of the lead SNP (chr9:g.35920172A > G) presented a 9.85% increase in birth weight (3.35 kg vs. 3.68 kg; Δ = 0.33 kg). Notably, the derived allele of this SNP exhibited low frequencies of < 0.1 across most global sheep breeds except Dorper, highlighting its potential for selective breeding applications. Leveraging lcWGS data, haplotype-based fine-mapping prioritized three candidate causal variants. A secondary QTL on chromosome 6 colocalized with the FecB mutation, a well-established locus associated with increased litter size. Intriguingly, individuals carrying one FecB allele showed a 6.18% reduction (0.22 kg) in birth weight, which tentatively indicates potential pleiotropic influences on both growth and reproductive traits.

**Conclusion:**

This study demonstrates the utility of lcWGS as a cost-effective, high-resolution tool for dissecting complex traits in livestock. Our findings not only advance the understanding of birth weight genetics in sheep but also offer a blueprint for accelerating genetic improvement programs in global livestock production through cost-effective, genome-wide approaches.

**Supplementary Information:**

The online version contains supplementary material available at 10.1186/s40104-025-01251-4.

## Background

Birth weight holds substantial economic significance in the realm of livestock production, playing a crucial role in the survival and ensuing growth of young animals [1]. It is well-documented that offspring born with lower weights face greater mortality risks, slower developmental rates, and inferior quality of meat [2, 3]. As a result, birth weight is deemed a key factor in breeding strategies that target enhanced productivity. A considerable genetic basis underlies this trait, as evidenced by moderate to high heritability scores [4–6]. Therefore, pinpointing the genes that affect birth weight is pivotal for deciphering the genetic and physiological mechanisms governing this trait, potentially leading to improved breeding selection methods and better growth outcomes in livestock.

Despite extensive research into the genetic basis of birth weight in sheep, universally recognized causative genes and quantitative trait loci (QTLs) remain limited, with most findings associated primarily with overall body weight. The pleomorphic adenoma gene 1 (*PLAG1*) has been reported as a major gene affecting cattle body size and weight [7], but its effect on the growth traits of sheep has yet to be elucidated. Another remarkable gene in sheep involves the ligand dependent nuclear receptor corepressor like (*LCORL*) locus, which is linked to body weight and stature through a genome-wide association analysis (GWAS) of 1,781 Australian Merino sheep [8]. Moreover, causal mutations remain scarce, with one notable exception being a genetic variation in the 3'untranslated region (3'UTR) of the myostatin (*MSTN*) gene, which inhibits translation, fostering muscle development or hyperplasia in Texel sheep [9]. The scarcity of identified causative genes and variants to this point can be attributed to small sample sizes and the complex genetic structure of these traits, characterized by minor effect sizes that lessen the impact of GWAS in revealing significant associations [10–12].

Identifying causal mutations for growth traits remains a major challenge in farm animals. The relatively short domestication period of approximately 11,000 years [13] for livestock, coupled with small effective population sizes, has resulted in extensive linkage disequilibrium (LD) around selected loci [14]. Moreover, previous studies in sheep have relied on a medium-density 50K SNP array with sparse genetic markers [15]. Recent developments underscore the advantages of lcWGS [16]. With sequencing depths typically 1× or lower, whole genome variants can be obtained with high accuracy by advances in imputation approaches [17–19]. Furthermore, lcWGS is considerably more cost-effective than low-density SNP chips are, allowing for the genotyping of larger populations within the same budget [16]. This increased sample size enhances the statistical power of GWAS [20].

In this study, we performed a GWAS to identify QTLs associated with body weight using lcWGS data from 3,007 Hu sheep lambs. We also fine-mapped potential causal SNPs and candidate genes through haplotype analysis and expression quantitative trait locus (eQTL) mapping. Our results identified the major QTLs affecting birth weight and demonstrated the superiority of lcWGS for large-scale GWAS and fine mapping of functional variants in livestock.

## Methods

### Animals and phenotypic measurement

The data used in this study included phenotypic records from 3,007 Hu sheep lambs reared at the Yulin Shanghe Farm in Shaanxi Province, China. This cohort included 1,547 males and 1,460 females, predominantly twins and triplets. The birth weights of these lambs averaged 3.30 kg and followed a normal distribution (Additional file 1: Fig. S1). Ear tissues were collected from each individual and stored at −20 °C for subsequent analysis.

### Whole-genome sequencing and imputation of lcWGS data

DNA was extracted using the standard phenol-chloroform method [21]. Paired-end sequence data for 3,007 individuals were generated using the DNBSEQ T7 platform (PE150), with an average sequencing depth of 1.24 ×. Low-quality reads were filtered out using fastp v0.23.2 [22] with default parameters. The clean reads were then aligned to the sheep reference genome ARS-UI_Ramb_v2.0 employing BWA-MEM v0.7.17 [23]. The conversion and sorting of BAM files were facilitated by SAMtools v1.16.1 [24]. Duplicate reads were identified and excluded using PICARD MarkDuplicates (http://broadinstitute.github.io/picard/) [25]. Genotype imputation was conducted using GLIMPSE v2.0.0 [17] with an internal haplotype reference panel and individual BAM-format alignment files, leveraging a probabilistic model optimized for low-coverage sequencing data to efficiently integrate haplotype information and sequencing likelihoods for accurate genotype inference. The resulting BCF files were then converted to VCF format with BCFtools v1.16 [26]. The genotype data were filtered using PLINK v1.90 [27] on the basis of two criteria: minor allele frequency (MAF) < 0.01 and *P* < 1 × 10⁻⁶ for HWE deviation. The accuracy of the whole chromosome imputation was assessed using a methodology we previously delineated [19]. For this purpose, the genotypes from 20 individuals, sequenced at an average depth of 27×, served as the reference set. To simulate a low-coverage scenario, SAMtools was employed to downsample the BAM files from these individuals to 1× coverage. The imputed genotypes were then juxtaposed with the reference genotypes to ascertain the concordance rate and the squared correlation (*r*^2^), providing a measure of the imputation accuracy. SNPs on the sex chromosomes were excluded from GWAS analyses but retained for heritability estimation.

### Estimation of birth weight heritability

We estimated the heritability (*h*^2^) of the birth weight based on the genetic relationship matrix (GRM) using restricted maximum likelihood (REML) analysis implemented in HIBLUP v1.5.3 [28]. The estimation model is as follows:

1 $$y=X\beta +Z\mu +Wm+e$$

where *y* is a n-vector of phenotypic observations for *n* individuals; *β* is a vector of fixed effects (covariates) including sex, litter size, parity and the first three principal components (PCs) of the genotype generated by PLINK v1.90; *μ* is the vector of additive genetic effects following a distribution of *N*(0, ${G\sigma }_{g}^{2}$), where *G* is GRM and ${\sigma }_{g}^{2}$ is the additive genetic variance; *m* is the vector of maternal environmental effects following a distribution of *N*(0, ${M\sigma }_{m}^{2}$), where *M* is the maternal identity matrix and ${\sigma }_{m}^{2}$ is the maternal environmental variance; *e* is the vector of residual effects following a distribution of *N*(0, ${I\sigma }_{e}^{2}$), where I is an identity matrix and ${\sigma }_{e}^{2}$ is the residual variance. *X*, *W* and* Z* serve as incidence matrices for *β*, *μ* and *m* respectively. The h^2^ was estimated as $\frac{{\sigma }_{g}^{2}}{{\sigma }_{p}^{2}}$, where ${\sigma }_{p}^{2}({\sigma }_{g}^{2} +{ \sigma }_{m}^{2} + {\sigma }_{e}^{2})$ is the phenotypic variance.

### GWAS of birth weight

GWAS analysis for birth weight traits of Hu sheep lambs was conducted using a linear mixed model in GEMMA v.0.98.5 [29]. To minimize potential sources of bias in the analyses, we excluded SNPs located on the sex chromosomes for GWAS. The estimation model is as follows:

2 $$y=X\beta +S\alpha +Zu+e$$

In the above equation, *S* is a design matrix of allele dosages for the SNPs, and *α* is the additive substitution effect. *μ* represents the vector of random effects in the model that follows the distribution *μ* ~ *N*(0, ${G\sigma }_{g}^{2}$), in which* G* was a genomic relationship matrix derived from all autosomal SNPs only; Other parameters are the same as those in model (1).

A genome-wide significance threshold of 5 × 10⁻⁸ was applied to identify significant SNPs [30–32]. Manhattan plots and quantile–quantile (Q-Q) plots were generated using the R package CMplot [33]. The identified SNPs were annotated using ANNOVAR [34].

### Regional haplotype analysis

The regions on chromosome 9 (chr9:35,899,774–36,017,682 bp) defined by high disequilibrium (*r*^2^ > 0.6) covering the lead SNP were analyzed. We then examined recombination events within the QTL using PLINK v1.90 [35], identifying two major recombination breakpoints and allowing us to narrow down the candidate interval to chr9:35,914,871–35,922,892 bp. The statistical significance of differences in birth weight between different groups was calculated using one-way analysis of variance (ANOVA), and *P*-values were adjusted for multiple comparisons using the Bonferroni correction.

### Statistical analysis

To test the differences in birth weight among different genotypes, a general linear model was used to adjust for sex and parity using R software (v.4.3.1), which minimizes the confounding effects of fixed factors. Multiple group comparisons were conducted via one-way analysis of variance (ANOVA), with *P*-values adjusted for multiple comparisons using the Bonferroni correction. All tests applied a statistical significance threshold of *P* < 0.05.

### eQTL mapping

Due to the tissue- and stage-specific expression pattern of *PLAG1*, and the low frequency of the candidate SNPs in global sheep populations except Dorper, adenohypophysis was the only tissue in which both *PLAG1* expression and sufficient SNP allele frequency allowed for effective eQTL analysis. We downloaded RNA sequencing data for 116 sheep adenohypophysis tissues from publicly available databases [36–39] (Additional file 2: Table S1). The data were first processed using Trimmomatic [40] to trim reads, ensuring quality and removing adapters. The cleaned reads were aligned to the ovine reference genome ARS-UI_Ramb_v2.0 using STAR v2.7.10b [41]. For transcript assembly and RNA-seq quantification, TPM (transcripts per million) values were obtained using StringTie v2.2.1 [42] and transcript counts were quantified using featureCounts v2.0.5 [43]. Our research focused on conducting *cis*-eQTL mapping to assess the genetic influences on gene expression within the adenohypophysis across 116 sheep individuals. We filtered the dataset to keep genes with TPM > 0.1 and transcript counts exceeding 6 in at least 20% of the samples. The gene expression matrix was normalized using TMM (trimmed mean of m-values) and then transformed into inverse normal values. *Cis*-eQTL mapping was conducted using a linear regression model implemented in TensorQTL [44]. The top five genotype PCs and the top seven phenotype PCs were included as covariates. We first performed *cis*-eQTL mapping in a permutation mode to calculate empirical *P* values and all the obtained *P* values were corrected by multiple-testing using false discovery rate (FDR) [45]. Subsequent analysis in nominal mode was used to identify the significant eGene-eSNP pairs, defining the empirical *P* value threshold corresponding to an FDR of 0.05.

### Transfection and luciferase assays

A luciferase reporter assay was used to measure the effects of the putative causal SNPs on transcriptional activity. For each SNP, a 25-bp fragment centered on the candidate mutation was artificially synthesized and subcloned and inserted into the pGL3-promoter luciferase reporter vector (Promega Corporation, Madison, WI, USA). The assays were performed using a dual-luciferase reporter assay system (Promega Corporation, Madison, WI, USA). 293 T cells were co-transfected with a firefly luciferase reporter construct containing different SNP alleles and a Renilla luciferase control vector using a Hieff Tans^TM^ Kit (YEASEN, Shanghai, China) according to the manufacturer's protocol. The transfected cells were rinsed with PBS and then lysed in 1× passive lysis buffer. The luciferase activity was measured 48 h post-transfection using the Dual-Luciferase Reporter Assay System. At least three independent triplicate experiments were performed. The mutant promoter activities were compared with those of empty vector and the wild-type promoter based on the normalized luciferase expression levels. Statistical analysis was performed, and the data are reported as the mean ± SD, with significance determined at *P* < 0.05.

## Results

### lcWGS and imputation accuracy validation

**Fig. 1:**
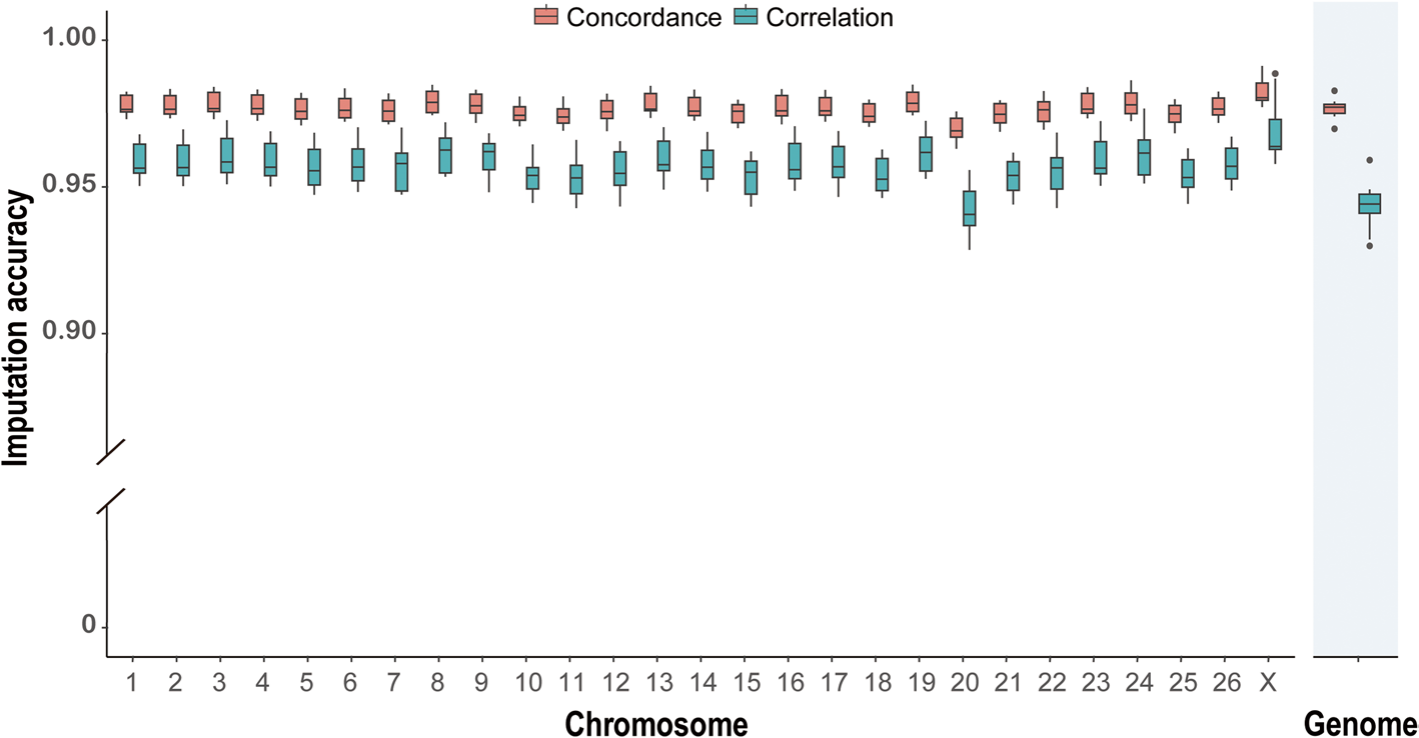
Imputation accuracy across whole chromosomes. A boxplot illustrating the Pearson correlation coefficients and concordance levels between true and imputed genotypes across various chromosomes for 20 individuals

The genomes of all 3,007 lambs were sequenced at an average depth of approximately 1.24× with a standard deviation of 0.27×. The sequencing depth across individuals ranged from 0.43× to 4.2×, with a median of 1.20× (Additional file 1: Fig. S2). The resulting low-coverage sequencing data were aligned to the reference genome (ARS-UI_Ramb_v2.0), and SNPs were imputed using GLIMPSE v2.0.0 [17]. This process identified 60,802,933 genetic variants across 27 chromosomes. After filtering out loci with a MAF less than 0.01 and those deviated from Hardy–Weinberg equilibrium (*P* < 1 × 10⁻⁶), 23,180,018 SNPs were retained for further analysis. The imputation accuracy was evaluated by comparing the imputed genotypes from lcWGS data with reference genotypes obtained from 27× WGS data of the same 20 Hu sheep individuals, following our previously reported strategy [19]. The concordance rate and squared correlation (*r*^2^), which measure imputation accuracy, were 97.8% and 94.5%, respectively (Fig. 1, Additional file 2: Table S2).

### Association analysis

Genetic and residual variances were estimated using genomic relationship matrices prior to conducting GWAS. The SNP-based heritability was estimated to be 0.38, when maternal effects were included.

**Fig. 2:**
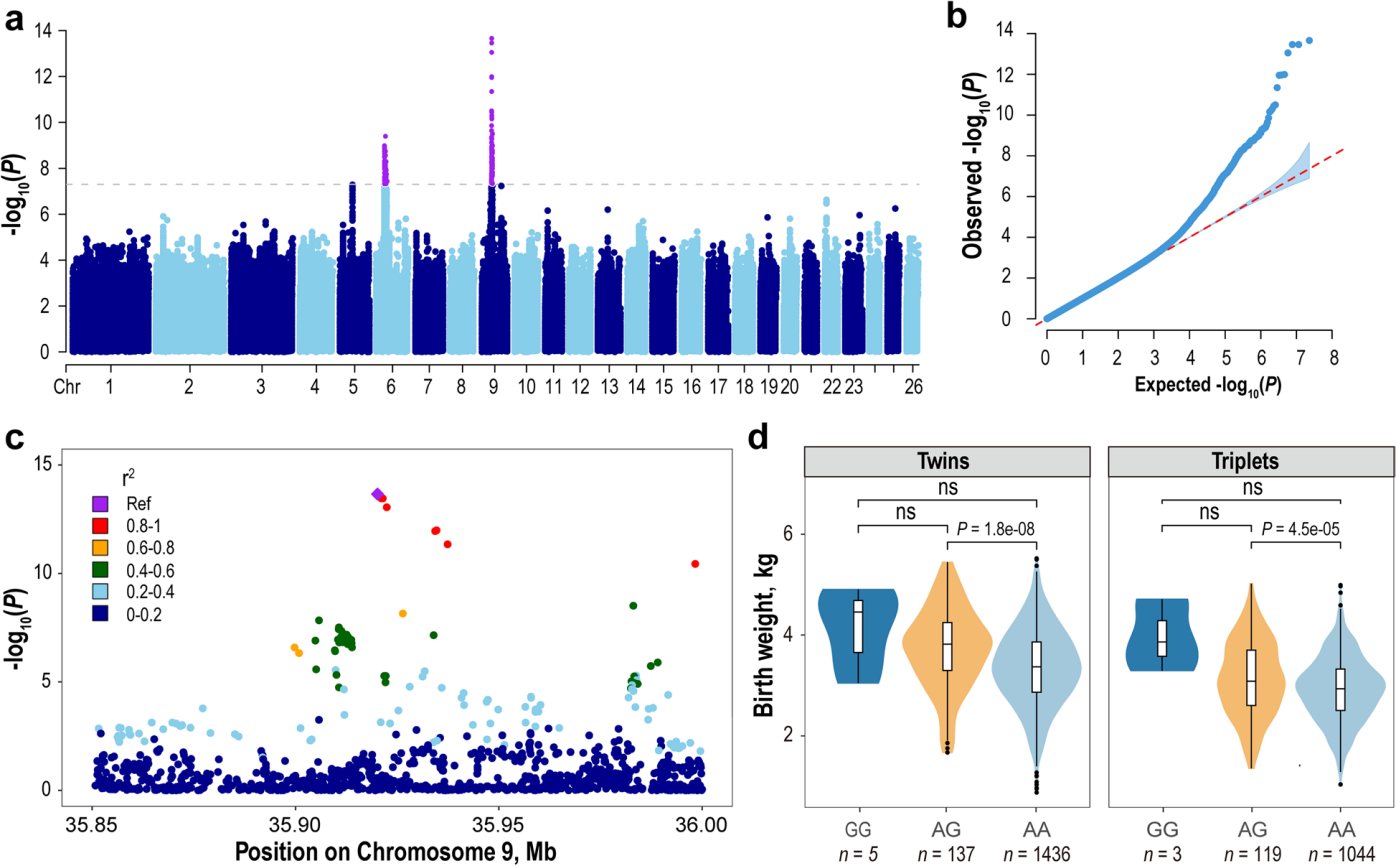
GWAS of birth weight in Hu sheep lambs. **a** Manhattan plots for GWAS of birth weight. The gray dashed lines denote the genome-wide significance threshold (5 × 10⁻⁸). **b** Q-Q plot of the GWAS results. **c** LocusZoom plot depicting the lead SNP and neighboring variants within a 150 kb region. **d** The differences in birth weight among individuals of three genotypes for the lead SNP

We performed a GWAS between imputed whole-genome variants and birth weight traits using a mixed model approach, identifying two major QTLs containing 181 significant SNPs (Additional file 2: Table S3). One QTL was mapped to chromosome 9, encompassing 106 significant SNPs within the 9:34.97–38.94 Mb interval. The Q-Q plot demonstrated a notable deviation from the expected distribution under the null hypothesis (λ = 1.002), indicating a robust association between this QTL and birth weight. The most significant SNP (*P* = 2.19 × 10^–14^) was an intronic variant (g.35920172A > G) within the *XKR4* gene and displayed high disequilibrium (*r*^2^ > 0.8) with another eight SNPs within a 97.5 kb region (chr9:35,920,172–36,017,682 bp, Fig. 2c). Although the number of homozygous mutant individuals was limited, this locus exhibited substantial effects on birth weight. On average, compared with their wild-type homozygous counterparts (3.35 kg), heterozygous individuals exhibited a significantly greater birth weight of 3.68 kg, corresponding to a 0.33 kg increase (9.85% relative improvement, *P* = 4.89 × 10^–12^) (Fig. 2d). When stratified by birth type, compared with wild-type AA homozygotes, heterozygous AG lambs exhibited a greater increase in body weight compared to wild-type AA homozygotes, with a more pronounced difference in twin-born individuals (AG: 4.01 kg vs. AA: 3.62 kg; Δ = 0.39 kg, *n* = 1,578) than in triplet-born lambs (AG: 3.34 kg vs. AA: 3.08 kg; Δ = 0.26 kg, *n* = 1,166). In addition, the derived G allele had a low frequency of 0.05, categorizing it as a low-frequency variant in the studied population.

**Fig. 3:**
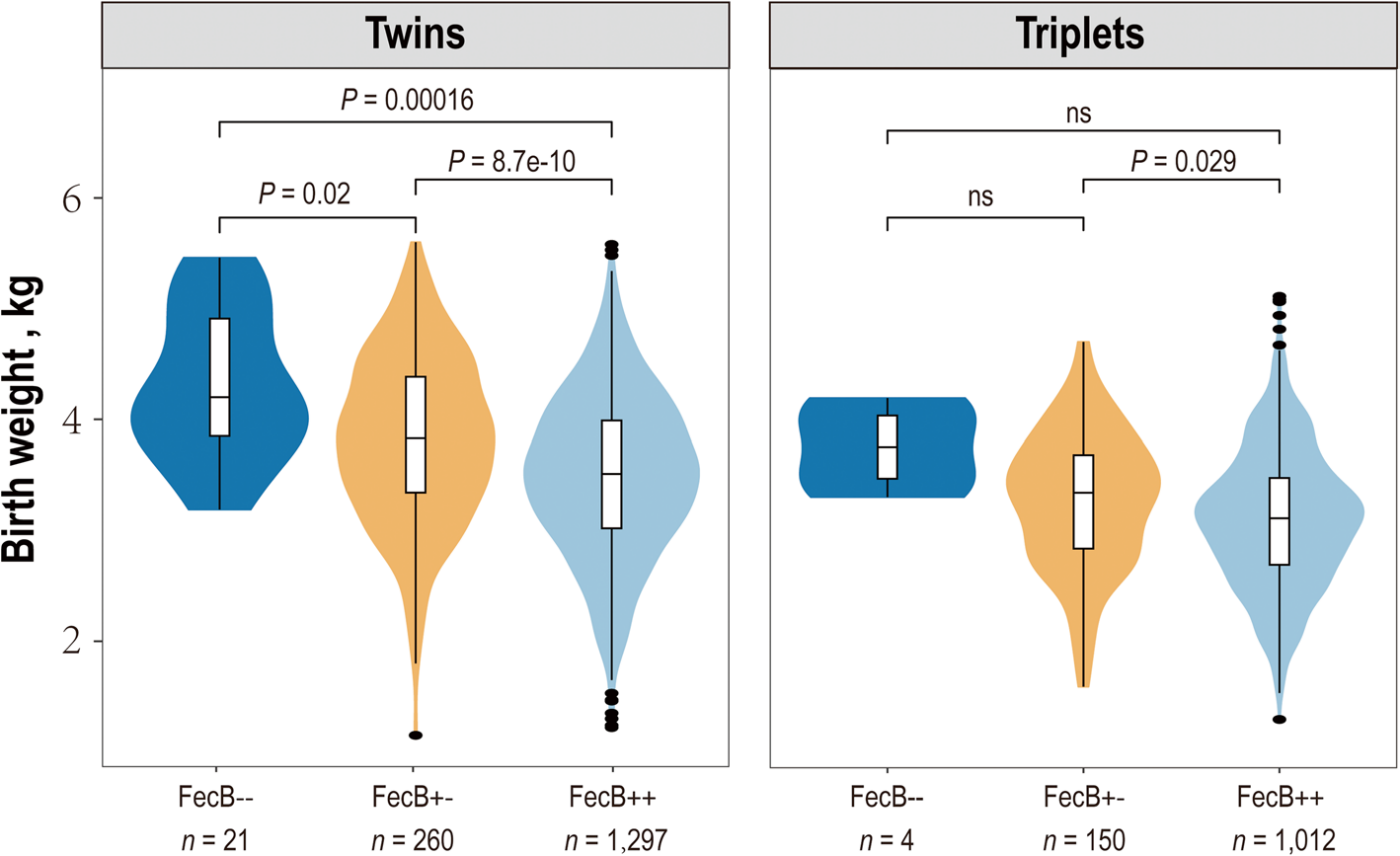
Birth weight differences among individuals with three FecB genotypes

Another significant peak was detected on chromosome 6 encompassing 75 significant variants across multiple genes within the chr6:30.01–35.95 Mb interval, including bone morphogenetic protein receptor type 1B (*BMPR1B*), UNC-5 netrin receptor C (*UNC5C*) and coiled-coil serine rich protein 1 (*CCSER1*). Among these significant SNPs, the most significant SNP (*P* = 4.02 × 10^–10^) was an intronic variant (g.33707226 A > C) within the *CCSER1* gene. However, we noticed that the well-documented FecB mutation (Q249R), known to affect ovine litter size [46], ranked 14^th^ (*P* = 3.31 × 10^–9^). Research has indicated that increased litter size is negatively associated with birth weight [47], but whether FecB mutation can directly affect fetal growth is not clear. To explore the direct impact of FecB mutation on birth weight, covariates such as litter size and sex were controlled in the GWAS to offset their potential confounding effects, confirming a significant, direct and negative association between FecB mutation and birth weight. Owing to the strong selection of FecB mutation in Hu sheep for increased litter size [47], homozygous FecB-- individuals were rare (*n* = 25) and thus excluded in following analysis. On average, homozygous FecB + + individuals exhibited a significantly lower birth weight (3.34 kg) compared to FecB+- counterparts (3.56 kg), which represents a 0.22 kg decrease (6.18% relative reduction, *P* = 1.43 × 10^–10^). When stratified by birth type, FecB + + individuals showed significantly lower body weights than FecB+- carriers in twin-born lambs (3.59 kg vs. 3.87 kg; Δ = −0.28 kg, *n* = 1,578), although this difference diminished in triplet-born lambs, where FecB + + individuals still exhibited a slightly greater decrease than their FecB+- counterparts (3.08 kg vs. 3.22 kg; Δ = −0.14 kg, *n* = 1,166) (Fig. 3).

### Fine mapping of putative causal variants within the QTL on chromosome 9

**Fig. 4:**
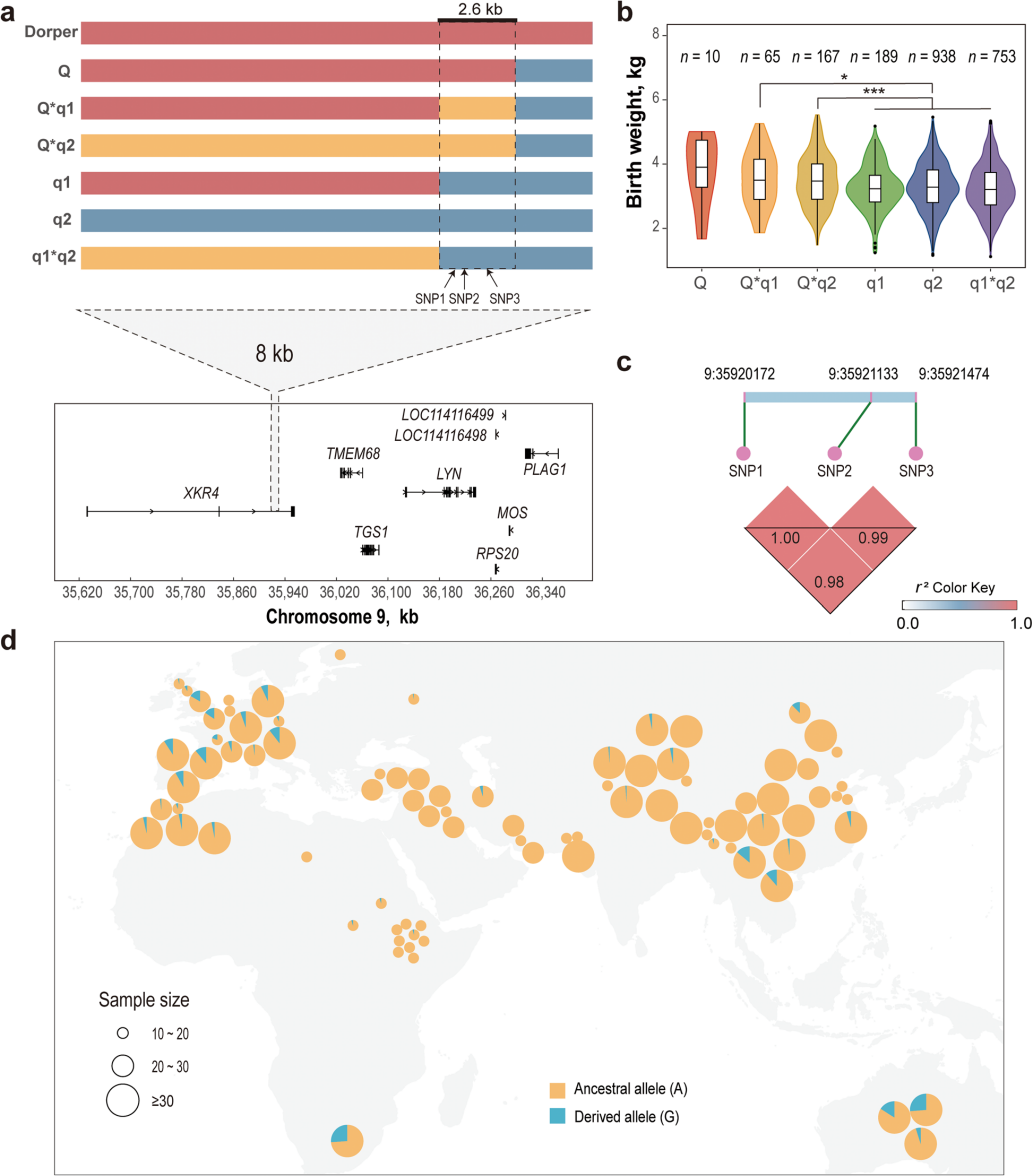
Fine mapping of the regulatory region for the QTL on chromosome 9. **a** An 8 kb interval delineated by recombination breakpoints. The dashed lines represent the breakpoints. The red and blue bands indicate homozygous haplotypes, whereas the yellow band indicates heterozygous haplotypes. Q represents haplotypes carrying the derived alleles, and q1 and q2 for noncarrier haplotypes. **b** Comparison of birth weights between different haplotypes. Bonferroni correction for multiple testing was used to adjust *P* values. ^*^P < 0.05, ^**^P < 0.01 and ^***^P < 0.001. **c** LD heatmap displaying pairwise *r*^2^ values among the three SNPs. **d** Distribution of derived allele frequencies for the lead SNP in sheep populations worldwide. The detailed frequency for each population is provided in Additional Table S4

To pinpoint the causative *cis*-regulatory locus on chromosome 9, we analyzed recombination events within the QTL among the 3,007 individuals in our study. This examination yielded two recombination breakpoints, thereby classifying the study population into six major haplotypes (Fig. 4a). Subsequent association analyses demonstrated a significant body weight advantage in the Q haplotype carriers relative to the noncarriers (Fig. 4b). The identified recombination breakpoints facilitated the delineation of causal variants to a concise 2.6 kb region on chromosome 9 (chr9:35,919,596–35,922,205). By analyzing allele frequencies in this region and employing a reference panel comprising global sheep breeds (http://animal.omics.pro/code/index.php/SheepVar), we found that the Q haplotype was also present in Dorper sheep. Within the haplotype, three SNPs were identified: the lead SNP (g.35920172A > G, hereafter designated SNP1) and two additional SNPs (SNP2: g.9:35921133G > T and SNP3: g.9:35,921,474 T > C) in complete linkage disequilibrium (Fig. 4c). These variants are absent in the ancestral mouflons, suggesting that they are derived mutations arising in domestics. Notably, the lead SNP displayed moderate or low allele frequency distributions in select breeds (Dorper: 0.38; Australian White: 0.16; Coopworth: 0.26; Texel: 0.16) while demonstrating effective absence (MAF < 0.1) across the 70 additional global sheep populations analyzed (Fig. 4d; Additional file 2: Table S4).

**Fig. 5:**
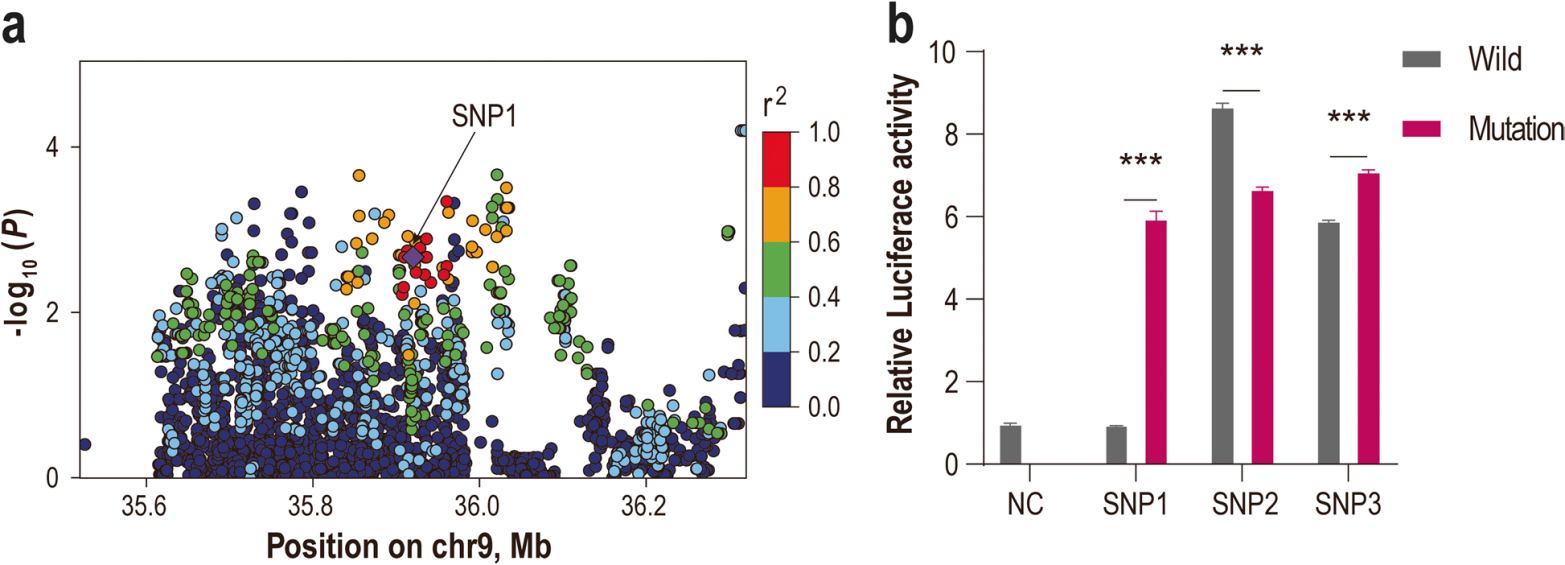
Functional validation of the three candidate SNPs. **a** Association of the lead SNP (denoted by purple diamond) with the expression of the *PLAG1* gene using eQTL mapping. **b** Luciferase reporter assay for the three candidate SNPs. ^***^P < 0.001

Although the lead SNPs are located within an intronic region of *XKR4* (XK-related protein 4) and approximately 390.4 kb upstream of *PLAG1*, several lines of evidence indicate that they are more likely to regulate *PLAG1* expression than that of other nearby genes. First, the lead SNP exhibited a MAF of 0.24 from adenohypophysis tissues, providing sufficient statistical power for eQTL mapping. We conducted *cis*-eQTL mapping for eight genes within a 1-Mb window: *LOC121820308*, *LOC101114620*, *XKR4*, *TMEM68* (transmembrane protein 68), *TGS1* (trimethylguanosine synthase 1), *LYN* (LYN proto-oncogene, Src family tyrosine kinase), *RPS20* (ribosomal protein S20), and *PLAG1* (Additional file 1: Fig. S3). The lead SNP and two highly linked variants showed the strongest and most significant association with *PLAG1* expression (Fig. 5a), whereas associations with *XKR4*, *TMEM68*, *LYN*, and other genes were weak or nonsignificant. Second, *PLAG1* is a well-characterized regulator of growth traits across livestock species, acting via *IGF2* signaling [7, 48], which provides strong functional support for its prioritization. Concurrently, our observations revealed that SNP1 strongly correlated with alterations in the expression of the proximal *TMEM68* gene (Additional file 1: Fig. S3). These findings suggest that these three SNPs could also modulate the expression of *TMEM68*, implicating a multifaceted mechanism of genetic influence on growth traits in sheep.

To determine whether these three SNPs reside within regulatory elements, we performed luciferase reporter assays (Fig. 5b). Cells transfected with constructs containing the derived alleles showed significantly elevated luciferase activity (*P* < 0.001), indicating that the region harboring these SNPs likely acts as a regulatory element. Notably, both the derived alleles of SNP1 and SNP3 increased luciferase activity compared with their wild-type counterparts, with SNP1 exhibiting a more pronounced effect than does SNP3. In contrast, compared with the wild-type allele, the SNP2 derived allele decreased luciferase activity. These findings suggest that while SNP1 and SNP3 may increase target gene expression, which is consistent with the anticipated increase in *PLAG1* expression associated with fetal growth, SNP2 may exert an inhibitory effect. The substantial impact of the SNP1 derived allele suggests that it may be a causal variant altering transcription factor binding affinity, thus modulating enhancer activity and upregulating gene expression.

## Discussion

In this study, we identified QTLs and candidate genes associated with birth weight in sheep using lcWGS of 3,007 individuals, which helps to increase the efficacy of selection strategies to improve sheep growth traits. To the best of our knowledge, this represents one of the largest WGS genotyping datasets assembled for a single sheep breed, comprising 60.80 million SNP markers. In addition to identifying a major QTL influencing birth weight, we demonstrated the feasibility and effectiveness of fine-mapping causal genes and variants using large-scale lcWGS in livestock populations. These findings expand our understanding of genetic mechanisms influencing growth traits, highlight the potential of the lcWGS approach for high-resolution GWAS in diverse sheep populations and underscore its considerable promise for advancing genomic prediction.

Despite numerous GWAS studies, the identification of major QTLs influencing sheep growth has been limited, with few reliable genes identified beyond the *MSTN* locus [11]. In this study, we conducted a large-scale GWAS for birth weight in Hu sheep and identified a significant QTL on chromosome 9 associated with this trait. Our eQTL analysis indicated that the identified locus may influence lamb growth by modulating the expression of *PLAG1*, a gene known to affect growth traits in various livestock species, including cattle and pigs [7, 49, 50]. Notably, a previously reported QTL associated with 180-day weight in Hu sheep [51] coincides with the genomic region identified in our study, suggesting that the QTL could influence weight across multiple developmental stages. While our hypothesis posits that the identified QTL region influences *PLAG1* expression, the eQTL mapping was based on RNA-seq data from adenohypophysis tissue, which may not fully capture gene expression dynamics relevant to growth such as fetal muscle or liver. However, adenohypophysis was the only available tissue in which both *PLAG1* expression and sufficient allele frequency of the lead SNP were detected. Future eQTL analyses in growth-relevant tissues, particularly fetal muscle or liver, along with experimental validation efforts, will be essential to further confirm the regulatory role of this locus. In addition, although luciferase assays in 293 T cells provided evidence for the regulatory activity of the candidate variants, the use of a heterologous human cell line may not accurately reflect ovine-specific transcriptional regulation. Thus, these findings should be interpreted with appropriate caution and warrant future validation in ovine-specific models. Furthermore, such studies would help clarify whether this QTL also regulates other proximal genes, such as *TMEM68*, contributing to a more comprehensive understanding of its role in growth and development in sheep.

Moreover, the favorable allele of the lead SNP (chr9:g.35920172A > G) was found at a relatively high frequency in Dorper sheep (allele frequency = 0.38), a globally prominent meat breed widely introduced to improve growth traits in local populations through crossbreeding [52]. In China, for example, Dorper bloodlines have been incorporated into the development of several new composite breeds—such as Huang-Huai Meat sheep [53] and Dumeng sheep [54]—designed to improve growth performance. This makes the identified SNP a valuable candidate for marker-assisted selection (MAS) in breeding programs that incorporate Dorper or Dorper-derived sires. Although the derived allele remains rare in most other global sheep breeds, targeted selection of sires carrying the desired allele could facilitate genetic improvement of birth weight and early growth traits. The discovery of this SNP thus provides important insight for future utilization of Dorper genetics to improve indigenous breeds through molecular breeding strategies.

Our study revealed an association between the FecB mutation on lower birth weight, suggesting a potential dual role of the *BMPR1B* gene in both body development and reproductive traits. Whether this mutation directly affects birth weight has been a subject of debate [55–57], as lambs carrying the FecB mutation are often born into large litters, raising questions about whether their lower birth weight is due to litter size effects or a direct negative impact of the FecB locus on growth [58, 59]. By focusing exclusively on twin-born lambs (thereby controlling for litter size as a confounding factor), we observed that FecB ++ carriers still presented significantly lower birth weights compared to noncarriers did. This finding points to a potential direct effect of the FecB mutation on fetal growth and aligns with previous studies [55]. FecB mutation causes amino acid changes (Q249R) to the *BMPR1B* gene, a receptor in the TGF-β superfamily that mediates the *BMP* signaling pathway [60]. This pathway is critical for various biological processes, including embryonic development, cellular differentiation, bone and cartilage formation, and reproductive function [61, 62]. The *BMPR1B* gene plays a pivotal role in skeletal and cartilage development, particularly in chondrocyte differentiation and bone growth [6, 63], underscoring its broad functional significance. This duality of FecB mutation might explain, from an evolutionary perspective, how it increases prolificacy without causing birthing complications. While these findings imply that *BMPR1B* may influence fetal growth in addition to its reproductive functions, causality remains uncertain. Functional validation studies are needed to clarify the molecular pathways by which the FecB mutation may influence fetal development.

The employment of lcWGS in our study was instrumental in effectively mapping QTLs and identifying potential causal mutations. By significantly reducing sequencing costs, lcWGS facilitated the genotyping of 3,007 individuals, generating a large dataset of genome-wide SNPs. This demonstrates that sequencing a large cohort at low coverage can yield more profound insights than high-coverage sequencing of fewer individuals or SNP arrays with limited markers [64]. In line with this, the breadth of sample size and haplotype diversity has been shown to be more critical than sequencing depth for achieving high genotype accuracy at segregating sites and increasing the power of association studies [15, 65]. Compared with array-based genotyping methods, lcWGS has shown superior capabilities for trait mapping in human studies [66, 67]. This approach significantly reduces genotyping costs while offering comprehensive SNP data with high imputation accuracy, facilitating the discovery of causal variants [16]. As a result, lcWGS holds substantial potential for advancing GWAS and genomic selection in livestock populations. However, its application in livestock genetics remains relatively limited [19]. Notably, lcWGS-based genotype imputation relies heavily on the availability of a high-quality reference panel [68], which is currently not publicly available for sheep. In this study, we utilized an internal reference panel for Hu sheep, achieving an imputation accuracy exceeding 98%. Nevertheless, the applicability of this panel to other sheep breeds requires further validation. We anticipate that the ongoing development of expanded sheep reference panels will further increase the utility of lcWGS, driving advancements in sheep genetics and breeding.

## Conclusions

The present study demonstrated that lcWGS data, coupled with imputation using a reference panel, achieves high imputation accuracy and enables GWAS on a large-scale population basis. We illustrated that lcWGS-based GWAS not only enhances mapping resolution and detection power but also aids in identifying causal mutations. We identified two major QTLs that are associated with birth weight in sheep and propose that the well-known FecB mutation may influence fetal growth. Our findings provide a framework for the application of lcWGS in sheep and potentially other livestock species.

## Supplementary Information

Additional file 1: Fig. S1. Birth weight distribution of twin and triplet lambs. Fig. S2 Distribution of autosomal sequencing depthacross samples. Fig. S3. eQTL mapping of eight genes surrounding the lead SNP.

Additional file 2: Table S1. RNA-seq download data information. Table S2. Evaluation of imputation accuracy by chromosomes. Table S3. Functional annotation of significant GWAS SNPs. Table S4. The distribution of derived allele frequencies for the lead SNP in worldwide sheep populations.

## Data Availability

The lcWGS data generated during the current study are not publicly available because low-coverage data can be used only after genotype imputation, and because of the large sample size of this study. However, the lcWGS data and VCF files are available from the corresponding author upon reasonable request. The summary statistics of GWAS is provided in Zenodo (https://doi.org/10.5281/zenodo.15696310).

## Abbreviations

ANOVA: Analysis of variance
BMP: Bone morphogenetic protein
BMPR1B: Bone morphogenetic protein receptor type 1B
CCSER1: Coiled-coil serine-rich protein 1
eQTL: Expression quantitative trait locus
FDR: False discovery rate
FecB: Booroola fecundity gene
GWAS: Genome-wide association study
LcWGS: Low-coverage whole genome sequencing
LCORL: Ligand dependent nuclear receptor corepressor like
LD: Linkage disequilibrium
LYN: LYN proto-oncogene, Src family tyrosine kinase
MSTN: Myostatin
PLAG1: Pleomorphic adenoma gene 1
QTLs: Quantitative trait loci
RPS20: Ribosomal protein S20
TMEM68: Transmembrane protein 68
TPM: Transcripts per million
TGS1: Trimethylguanosine synthase 1
UNC5C: Unc-5 netrin receptor C
XKR4: XK related 4
