# Supplementary material for: Fine mapping genetic variants affecting birth weight in sheep: a GWAS of 3007 individuals using low-coverage whole genome sequencing
Source: J Anim Sci Biotechnol. 2025 Aug 12;16:115. doi: 10.1186/s40104-025-01251-4 (PMC12341293; doi:10.1186/s40104-025-01251-4)
Supplement: Supplementary file 1 — Additional file 1: Fig. S1. Birth weight distribution of twin and triplet lambs. Fig. S2 Distribution of autosomal sequencing depthacross samples. Fig. S3. eQTL mapping of eight genes surrounding the lead SNP. [file 40104_2025_1251_MOESM1_ESM.docx]

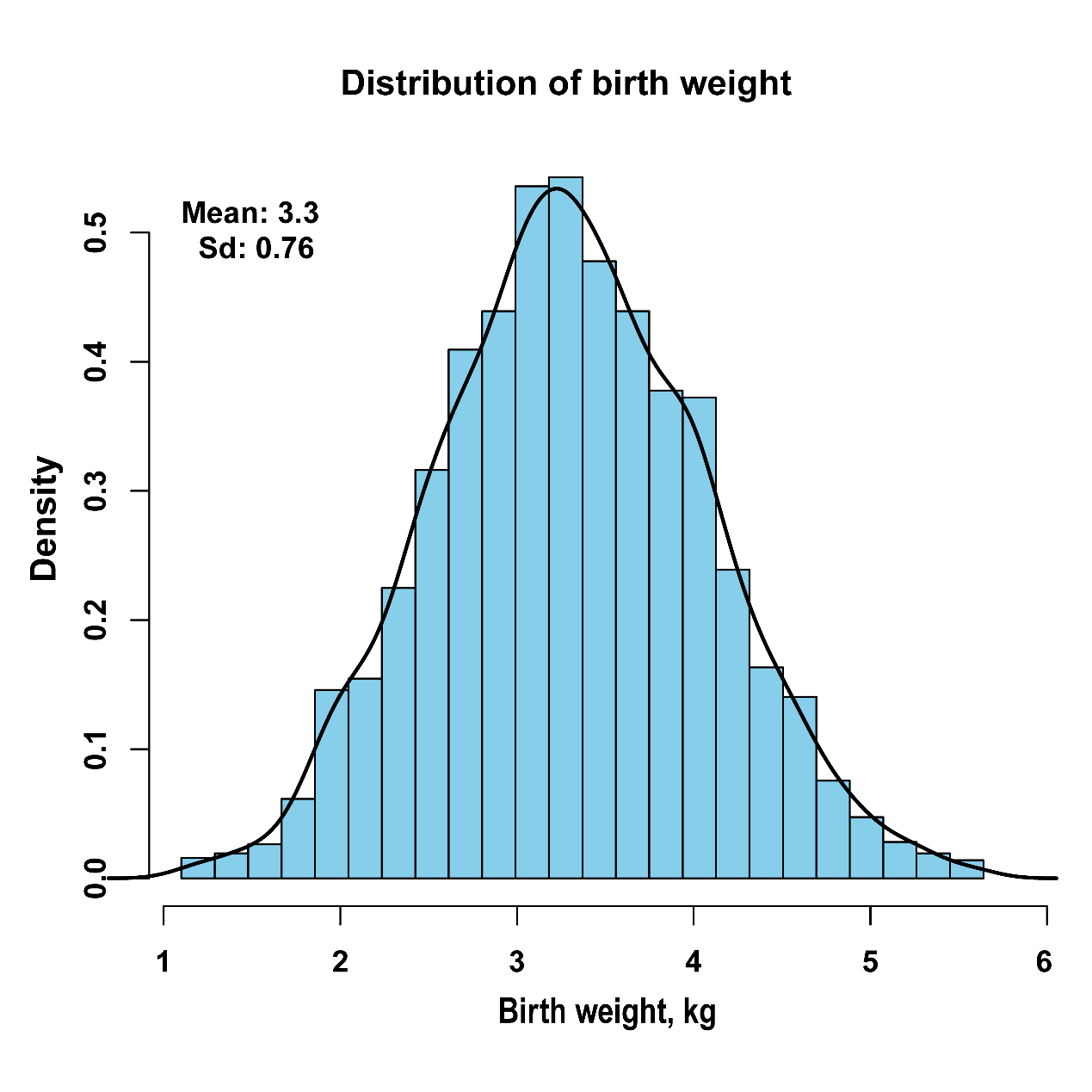


**Fig. S1** Birth weight distribution of twin and triplet Lambs


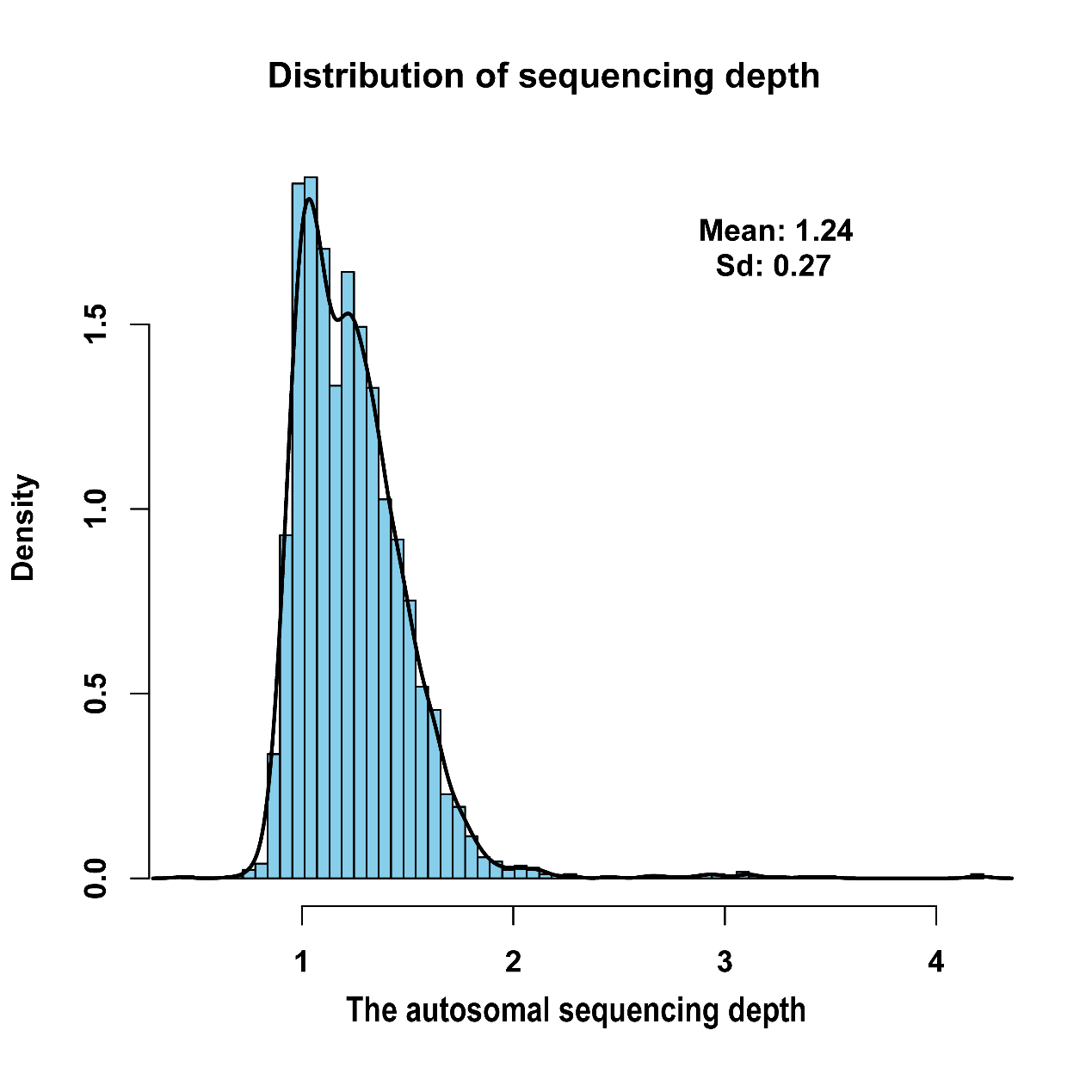


**Fig. S2** Distribution of autosomal sequencing depth (×) across samples


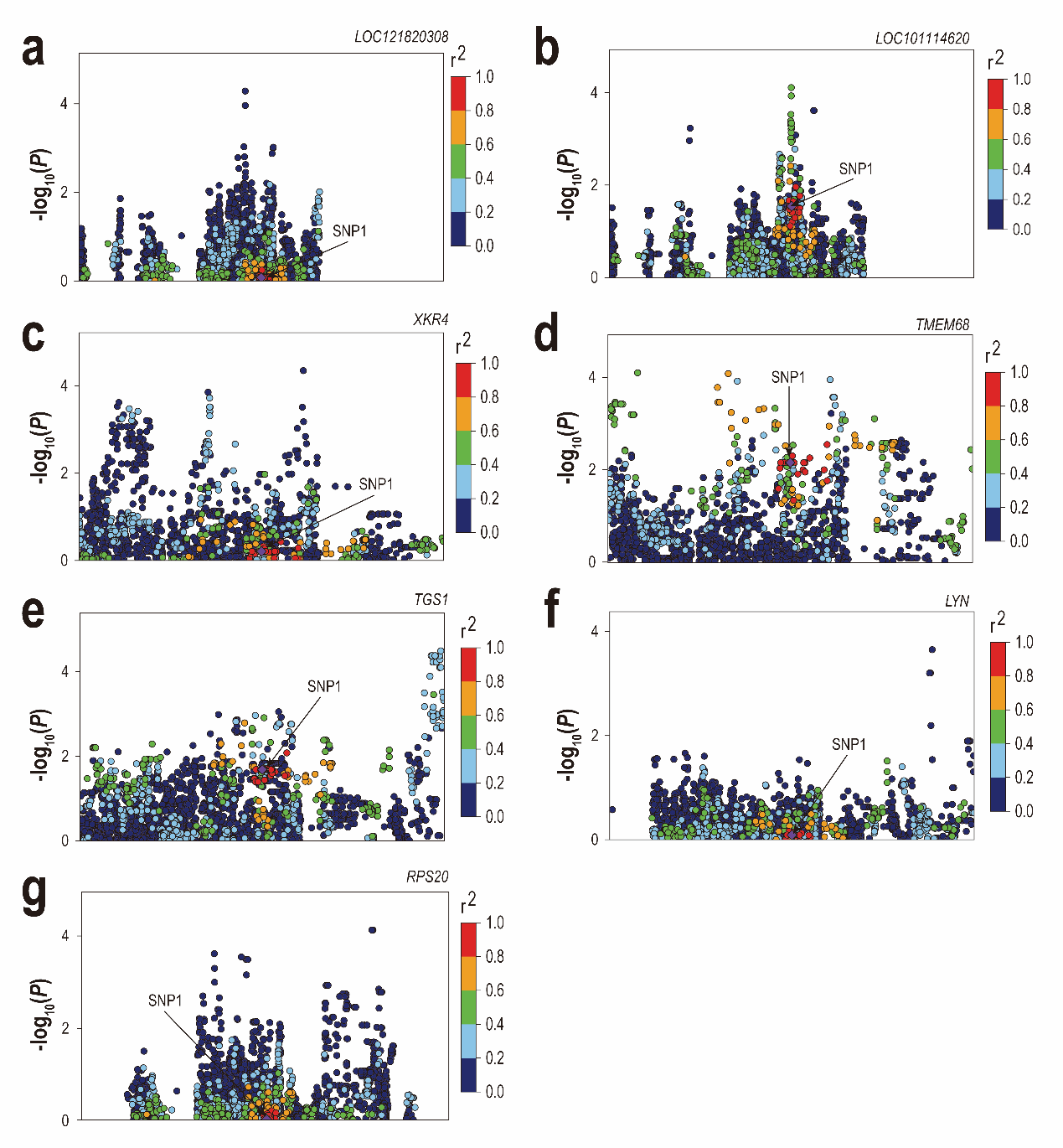


**Fig. S3** eQTL mapping of eight genes surrounding the lead SNP.
